# The collaboration code: how humans and AI work together across millions of conversations on job tasks

**DOI:** 10.3389/frai.2026.1801235

**Published:** 2026-07-14

**Authors:** Patrick D. Lynch

**Affiliations:** Hult International Business School, Cambridge, MA, United States

**Keywords:** augmentation and automation, generative AI adoption, human–AI collaboration, protection motivation theory, social exchange theory, socio-technical systems, technology acceptance model, post-adoptive behavior

## Abstract

As generative AI transforms work across all fields, understanding human–AI collaboration is critically important to the future of work. This study analyzed behavioral data from millions of Claude AI conversations mapped to occupational tasks via the O*NET taxonomy to examine whether human–AI collaboration patterns are consistent with expectations derived from traditional technology adoption frameworks. Utilizing Technology Acceptance Model, Protection Motivation Theory, Social Exchange Theory, and Socio-Technical Systems Theory as parallel lenses on post-adoptive behavior, the analysis examined how interaction patterns predict AI usage intensity, risk-management behaviors, and the development of cognitive partnerships. Results demonstrated that collaborative interaction patterns, including iterative refinement and learning-oriented exchanges, predicted significantly higher AI usage intensity, consistent with expectations derived from TAM. Contrary to Protection Motivation Theory predictions, risk-management behaviors such as validation and feedback loops were positively associated with usage intensity, suggesting these function as protective engagement strategies that enable continued use, not resistance mechanisms. Analysis of extended thinking mode usage suggested distinct trust pathways: delegation trust, where users rely on AI’s autonomous reasoning, versus collaboration trust, built through iterative dialogue, although sensitivity analyses indicate that task complexity may partly account for this divergence. A cross-level reversal emerged whereby augmentation patterns positively predicted usage intensity at the task level but negatively predicted user adoption at the cluster level, indicating that depth and breadth of AI use follow different dynamics. These findings challenge assumptions that caution suppresses AI use and suggest that effective human–AI partnership supports cognitive partnerships through multiple complementary pathways. For practitioners and leaders, supporting diverse ways of interacting with AI can be important for successful integration. The analyses drew on the Anthropic Economic Index dataset of 3,365 occupational tasks grouped into 593 task clusters, with model sample sizes varying by hypothesis.

## 1 Introduction

The future of AI-augmented work is being shaped one chatbot interaction at a time. Across millions of everyday exchanges between people and AI systems, people are learning to work with increasingly capable machines. And those machines, in turn, are reshaping human work. While much attention has focused on rapid technical advances, important questions remain about how people choose to work with these machines.

Traditional technology adoption research has provided valuable insights into user attitudes and system capabilities (Brynjolfsson and Mitchell, 2017; Binns, 2018; Dwivedi et al., 2019). This investigation centers on the actual experience of how people and AI learn to work together through millions of occupation-related chatbot interactions (Anthropic, 2025a). These conversations signal a new process where human behaviors, AI capabilities, and organizational structures are mutually adapting in ways that are only beginning to be understood.

This body of work has largely focused on whether users accept or reject a technology. This framing overlooks the relational and systemic dimensions that make some human–AI partnerships succeed while others fail. When AI functions as a cognitive partner, the nature of ongoing use matters as much as the initial adoption decision (Lynch et al., 2025a,b). Research is needed both on whether people adopt AI and the evolving character of human–AI relationships that are reshaping the nature of work.

Recently, concerns about the risks of AI have intensified, ranging from immediate issues such as factual inaccuracies, security vulnerabilities, and misuse to broader societal harms including disinformation, deskilling, and erosion of human agency. These concerns now command attention at a global scale. Yet focusing on risk alone misses how people adapt their behavior and continue to engage with AI by choice or directive.

This study addresses that gap by examining how people work with AI once it is already part of their tasks. It looks at how different interaction patterns, such as iterative refinement of chatbot requests, validation of responses, learning, and task delegation, relate to usage intensity and to the collaborative use that marks AI as a cognitive partner. Extending that perspective, the central question is whether observed behavior across millions of real-world AI conversations is consistent with the expectations derived from established theoretical frameworks.

Because the data capture behavior among users who have already adopted Claude, the study is positioned in the post-adoption phase of technology use (Jasperson et al., 2005; Bhattacherjee, 2001). It asks not why people adopt AI but how adopted use is engaged through working with AI on tasks, and at what intensity (Strong et al., 2014). The frameworks are lenses that generate expectations about these post-adoptive patterns, not as theories whose foundational constructs the behavioral data can directly test.

The contribution is both theoretical and empirical. Theoretically as lenses on post-adoptive behavior, four established frameworks generate the study’s expectations: the Technology Acceptance Model (TAM; Davis, 1989) for usefulness-driven engagement, Protection Motivation Theory (PMT; Rogers, 1975) for protective behavior under perceived risk, Social Exchange Theory (SET; Blau, 1964) for behavioral trust calibration, and Socio-Technical Systems theory (STS; Trist and Bamforth, 1951) for socio-technical alignment of automation and augmentation behaviors and cross-level consistency. Empirically, it examines whether behavior across millions of real-world conversations is consistent with the expectations derived from these lenses.

Hypotheses are examined using behavioral data from the Anthropic Economic Index (Handa et al., 2025), analyzing task-specific interactions mapped to the O*NET occupational taxonomy maintained by the U.S. Department of Labor. The data capture real-world interactions with Claude AI across a wide range of users and tasks, with prompts classified as automation (AI performing the task independently) or augmentation (AI helping a human complete the task). This enables analysis at both task and occupational cluster levels, spanning knowledge workers in technology, education, legal services, marketing, and administration. The data also capture the recently introduced extended thinking mode, where users explicitly request that the AI engage in deeper, more deliberative reasoning, offering a window into more effortful and complex human–AI engagement.

## 2 Theoretical framework

### 2.1 Frameworks for post-adoptive human–AI collaboration

The frameworks reviewed below were developed to explain technology acceptance, that is, the antecedents of an individual’s decision to adopt a new system. The present data capture how people who have already adopted Claude exercise that adoption across occupational tasks. Following Jasperson et al. (2005), these data are treated as records of post-adoptive behavior, defined as “the myriad feature adoption decisions, feature use behaviors, and feature extension behaviors made by an individual user after an IT application has been installed, made accessible to the user, and applied by the user in accomplishing his/her work activities” (p. 531). This distinction aligns with Bhattacherjee (2001) who showed that “acceptance and continuance are two temporally and conceptually distinct and possibly incongruent phases of IS [information systems] use” (p. 357) and respecified perceived usefulness as a post-use, confirmation-shaped belief rather than a pre-adoption expectation; subsequent work has continued to integrate acceptance logic into models of continued use (Tsai et al., 2020). The hypotheses are framed not as tests of whether people adopt AI, but as expectations about how post-adoptive usage unfolds among those who already use it.

While the study is focused on the post-adoption phase of AI use, the behavioral record does not contain the perceptual constructs (perceived usefulness, perceived risk, felt trust) that these frameworks posit. This is addressed by grounding the analysis in affordance actualization (Strong et al., 2014). Strong and colleagues define an affordance as “the potential for behaviors associated with achieving an immediate concrete outcome and arising from the relation between an artifact and a goal-oriented actor or actors” and actualization as “the actions taken by actors as they take advantage of one or more affordances through their use of the technology to achieve immediate concrete outcomes in support of organizational goals” (pp. 69–70). Claude affords distinct modes of work, including directive delegation, feedback-loop debugging, iterative refinement, learning-oriented exchange, output validation, and extended-thinking deliberation. The data record which of these affordances are actualized, in which tasks, and at what intensity. Because actualization is defined as goal-oriented action rather than as a perception of the technology, the behavioral measures are not proxies standing in for unobserved attitudes; the action itself is the construct. This is the sense in which large-scale interaction data are most informative: they document what users do with an adopted technology, not what they report believing about it.

Within this umbrella, the four frameworks function as parallel expectation-generating lenses rather than as competing theories under test, and each addresses a distinct behavioral dimension of post-adoptive actualization. Aligned with Bhattacherjee (2001), TAM anticipates that usefulness-driven engagement co-occurs with more intensive use (H1). PMT anticipates that risk-management behaviors coincide with reduced use (H2a–H2c). SET anticipates behavioral trust calibration in how users distribute reasoning between delegation and collaborative co-production (H3). STS anticipates that joint optimization of automation and augmentation tracks with deeper engagement at the cluster level (H4). Two further hypotheses combine lenses: a cross-level mediation linking TAM and SET (H5) and a cluster-level interaction linking SET and STS (H6).

### 2.2 The technology acceptance model

The Technology Acceptance Model (TAM), introduced by Davis (1989), proposes that people adopt new technologies when they believe a technology will improve their performance (perceived usefulness) and be relatively effortless to learn and use (perceived ease of use). Recent research confirms that TAM remains a valuable framework for understanding initial AI adoption decisions, though important extensions are emerging. Ibrahim et al. (2025) validated an extended TAM for AI adoption, finding that perceived usefulness remains the strongest predictor of attitudes toward AI use. However, the study also identified individual differences, including attitudes toward and beliefs about AI, that shape adoption and use. This research suggests that individuals’ internal perspectives about AI’s role in their development matter as much as the technology’s usability.

Shrivastava (2025) provides complementary evidence while highlighting TAM’s limitations when applied to generative AI. Examining college students’ adoption of generative AI, Shrivastava found when users perceive AI as useful, intuitive, and reliable, they are significantly more likely to adopt it and less likely to avoid it. However, the study also showed that trust in AI governance significantly enhances these perceptions, where regulatory confidence amplifies perceived usefulness. A user may find an AI tool highly useful yet still reject it due to privacy risks or ethical concerns that TAM does not address. A recent review of TAM in the adoption of healthcare innovations similarly found that boundary conditions matter: patient use of technology, including AI, depends more on ease of use, whereas perceived usefulness matters more for healthcare professionals (Lee et al., 2025).

Consider a student using an AI writing assistant: even if the tool produces high-quality output effortlessly (high perceived usefulness and ease of use), concerns about privacy, data security, or ethical issues like plagiarism detection may cause the student to avoid or resist it despite its clear benefits. This pattern suggests that TAM variables do not operate independently and that social-political context shapes how users evaluate AI’s functionality. Together, these findings indicate that TAM’s benefit-driven logic extends to AI, but only alongside individual differences such as AI mindset, risk perception, trust in governance, and expertise. This evidence, however, concerns the decision to adopt. The present data begin on the other side of that decision, among users already working with the tool.

From a post-adoptive behavior lens, TAM contributes one expectation to the present study: that usefulness-driven engagement, expressed as iterative refinement and learning-oriented exchange, co-occurs with more intensive use of an already-adopted tool. Perceived usefulness and ease of use are not observed directly; following Bhattacherjee (2001), they are treated as the post-adoption logic behind continued use rather than as measured antecedents of an adoption decision (H1).

### 2.3 The protection motivation theory

TAM addresses why people engage with useful technology, but not why they hesitate. Protection Motivation Theory (PMT), developed by Rogers (1975), addresses that gap by modeling how individuals respond to perceived risk. Under PMT, a threat appraisal combines the perceived likelihood of harm (perceived vulnerability) with its perceived seriousness (perceived severity). A parallel coping appraisal weighs whether protective measures work (response efficacy), whether the individual can implement them (self-efficacy), and whether the effort required is worthwhile (response cost).

Recent research on generative AI adoption found that for AI tools like ChatGPT, users are more concerned with the likelihood of problems occurring than with how severe those problems might be. Fu et al. (2025) found that perceived vulnerability (the probability of encountering misinformation, errors, or misuse) significantly decreased students’ and educators’ intention to use ChatGPT in academic settings, while perceived severity (how serious those errors might be) had no significant effect. This suggests that AI adoption is less about catastrophic failure scenarios and more about the everyday reliability and trustworthiness of the technology. Fu et al. (2025) found that when users believe they have the skills to use AI effectively (self-efficacy) and that protective strategies work (response efficacy), they are significantly more likely to adopt the technology despite perceived risks. Interestingly, the effort required to implement these protective strategies (response cost), such as fact-checking AI outputs or validating information, did not deter users, suggesting that people are willing to invest effort in risk management when they feel capable of doing so.

PMT frames the expectation that perceived risk elicits protective behavior that dampens use. Because the data record protective behaviors (validation and feedback loops) but not the perceptual PMT foundations (perceived vulnerability, severity, and coping efficacy), the lens is scoped here to behaviors: whether risk-management activities coincide with reduced use, as PMT would predict, or with continued use (H2a–H2c).

### 2.4 Social exchange theory

Neither TAM nor PMT alone explains why some users develop deep, trusting partnerships with AI systems while others maintain purely transactional relationships. Social Exchange Theory (SET) addresses this gap by examining how trust in governance and regulatory fairness shapes user decisions. Grounded in sociological norms of reciprocity (Homans, 1961; Blau, 1964; Ahmad et al., 2023), SET explains why some people are more willing to share personal information with organizations they trust. It distinguishes between reciprocal exchanges built on mutual trust (like social networking, where people share information expecting undefined future benefits such as social support) and negotiated exchanges based on formal agreements (like online shopping, where transaction terms are set in advance). Research shows that trust developed through reciprocal social interactions can spill over to increase willingness to share data, but when personal trust is absent, users rely on assurance structures of legal and regulatory frameworks like GDPR as a proxy for trust (Urbonavicius et al., 2021). Importantly, perceived regulatory effectiveness significantly increases willingness to share data even when users feel less personal control over their information, suggesting that can compensate when interpersonal trust is ambiguous.

For AI systems specifically, SET shows that trust in governance and regulatory fairness fundamentally shapes how users evaluate the exchange between themselves and AI technologies. Shrivastava (2025) found that trust in governance acts as a bridge between the usefulness and risk appraisals: a useful tool still meets resistance when users doubt it is managed responsibly, while credible governance lets users treat the tool as both safe and worth engaging deeply. Trust amplifies the value of functional benefits, creating a reinforcing cycle in which good experiences build trust, which in turn enables deeper engagement.

This mediation effect suggests that TAM and PMT operate through interconnected rather than independent pathways: functional benefits of AI alone cannot overcome resistance without adequate trust infrastructure, and risk mitigation requires more than technical solutions. However, individual engagement with AI occurs within larger organizational and occupational contexts that either support or constrain it.

As a lens, SET contributes expectations about behavioral trust calibration: how users distribute reasoning between delegation and collaborative co-production, and when they invest in extended deliberation. Felt trust is not measured; the behavioral patterns are read as consistent or inconsistent with these expectations. SET also informs two combination hypotheses: the cross-level mediation with TAM (H5) and the cluster-level interaction with STS (H6).

### 2.5 Socio-Technical Systems Theory

Socio-Technical Systems (STS) Theory technology integration requires both the technical system and the social system to work together (Trist and Bamforth, 1951). The technology must fit the actual tasks people do and align with how they work together, make decisions, and share authority. Recent research suggests that this requires constant adjustment. Amanollahnejad et al. (2026) studied how organizations adopt AI and found that technical changes immediately trigger social reactions, like shifts in who has authority or how workflows operate, which then require further technical adjustments in a continuous cycle. A manager in their study shared, “It’s never done. Every time we think AI is working, the business changes again” (punctuation added). This means successful AI integration is less about finding the perfect match between technology and organization, and more about maintaining the ability to keep adapting as both the technology and the work continue to evolve.

STS also helps explain why AI use can stall even when the technology works well and individuals want to use it. A capable tool can go underused when organizational structures, such as reward systems that still measure performance by billable hours rather than quality or client outcomes, work against it. STS shows that this kind of misalignment between what the technology enables and how the organization operates impedes sustained use. AI integration may falter not because the technology lacks capability or because people resist change, but because organizations struggle to maintain the ongoing work of aligning technical systems with social practices, reward structures, and decision-making processes. This ongoing condition requires continuous attention and negotiation (Amanollahnejad et al., 2026), which suggests identical tools succeed in some organizations and stall in others.

For this study, STS is applied behaviorally rather than to organizational alignment itself: the data cannot measure implementation context, reward structures, or organizational joint optimization. It yields two expectations using observed data: that balancing automation and augmentation at the cluster level is associated with deeper engagement (H4), and that patterns cohere across the task and cluster levels, a cross-level consistency that also underlies the interaction examined with SET (H6).

## 3 Method

### 3.1 Hypotheses

#### 3.1.1 Hypothesis 1 (technology acceptance model)

Handa et al. (2025) found, in an analysis of Claude.ai conversations, that 57% of AI usage involves augmentative patterns (task iteration, learning, validation) while 43% involves automation (directive execution, feedback-driven debugging). These descriptive findings establish baseline usage patterns, but they do not address whether such patterns relate to how intensively AI is used once committed to a task. Drawing on post-adoptive usage patterns associated with TAM, this hypothesis examines AI use intensity drawn from the Anthropic Economic Index data. Task-level analyses examine individual work activities (e.g., “Write and debug computer code”), while cluster-level analyses examine groups of related tasks that share similar patterns of AI usage (e.g., all software debugging activities).

TAM holds that perceived ease of use and perceived usefulness drive the decision to adopt a technology. In the post-adoptive setting examined here, the analysis examines conversation patterns classified by Tamkin et al. (2024). Task iteration captures conversations where users collaboratively refine outputs (e.g., “Good start, but can we add more detail?”), signaling perceived ease of use. Learning captures conversations where users seek understanding (e.g., “Can you explain how this works?”), signaling perceived usefulness as a knowledge partner. Usage intensity is measured as the proportion of all AI conversations mapped to each task.

*H1 (technology acceptance model)*: Tasks characterized by higher levels of iterative refinement (task_iteration) and learning-oriented interaction (learning) will show higher AI usage intensity (task_conversation_share).

#### 3.1.2 Hypothesis 2 (protection motivation theory)

PMT traditionally predicts that heightened perceived risk reduces technology adoption. When using AI chatbots, there are observable risk-management behaviors such as feedback loops and output validation. Feedback loops occur when users repeatedly correct AI errors (e.g., “That code gave me an error… now I’m getting a different error…”). Validation occurs when users verify AI outputs (e.g., “Are you sure the calculation is correct?” or “Can you check if my logic is sound?”). These may therefore function not as signals of avoidance, but as protective coping strategies that enable continued engagement under conditions of uncertainty.

This typically is observed as resistance or avoidance: users who perceive privacy, security, or performance risks disengage from the technology (Shrivastava, 2025). In this study, frequent error correction through feedback loops and explicit output validation are treated as actualized risk-management behaviors, capturing how users monitor and manage potential errors during interaction with AI rather than serving as a measure of perceived risk itself.

However, other studies suggest that when users have sufficient coping efficacy (belief in their ability to manage risks), threat perceptions do not necessarily suppress engagement but instead shape how users interact with AI systems (Fu et al., 2025). This distinction between usage intensity (how much AI is used) and engagement style (how AI is used) frames protective behaviors when engaging with AI that may outweigh threat perceptions in shaping behavior (Fu et al., 2025).

Some users may adapt their interaction style to retain oversight and control, shifting away from fully automated task execution toward more cautious, interactive use to complete tasks. This suggests that PMT may require reframing when applied to in-use human–AI collaboration, where perceived risk shapes how AI is used rather than whether it is used.

This study examines PMT-derived expectations through three complementary hypotheses that distinguish these mechanisms:

*H2a (protection motivation theory)*: Tasks characterized by higher levels of risk-management behaviors (feedback_loop + validation) will show lower AI usage intensity (task_conversation_share) reflecting threat-driven avoidance.

*H2b (delegation style)*: Among tasks where AI is used, higher risk-management behaviors will predict lower use of pure automation delegation (directive), reflecting protective strategies that avoid "black-box" processing where users lack visibility into AI reasoning.

*H2c (collaboration style)*: Among tasks where AI is used, higher risk-management behaviors will predict higher use of collaborative augmentation (task iteration), reflecting protective strategies that maintain human oversight through active co-creation.Together, these hypotheses test whether risk-aware behaviors manifest as reduced usage intensity (H2a) or as strategic shifts in interaction patterns that enable continued engagement under protective monitoring (H2b, H2c). If H2a is disconfirmed but H2b or H2c are supported, this would suggest PMT operates differently in ongoing use contexts, shaping engagement quality rather than suppressing engagement quantity.

#### 3.1.3 Hypothesis 3 (social exchange theory)

SET suggests that trust develops through reciprocal exchanges (Ahmad et al., 2023). When a chatbot user requests deeper deliberative reasoning from the AI by engaging extended thinking mode, it represents an investment in cognitive partnership and is read here as a behavioral indicator consistent with trust. This study predicts that collaborative work patterns, such as when users refine AI outputs through dialogue and seek conceptual explanations, will be associated with greater use of extended thinking mode. For example, a chatbot user who asks, “Can you make this more detailed? Now add examples,” is demonstrating collaborative trust. Just as people collaborate with trusted colleagues to think things through, users who trust AI will more readily ask it to engage in deeper deliberation. This willingness to request deeper AI reasoning is treated as consistent with established trust in the AI as a thinking partner. Because extended thinking may also reflect task complexity rather than trust, analyses control for technical domain, consistent with task-technology fit (Goodhue and Thompson, 1995).

*H3 (social exchange theory)*: At the cluster level, higher levels of collaborative augmentation (cluster_augmentation_index) will predict greater use of extended thinking mode (cluster_thinking_fraction).

#### 3.1.4 Hypothesis 4 (Socio-Technical Systems Theory)

STS suggests that successful technology use takes aligning what the technology can do and how people work (Amanollahnejad et al., 2026). When users have integrated AI into their work, sometimes delegating tasks and other times collaborating, they will use extended thinking for complex problems needing deeper deliberation. This study predicts that chatbot users effectively balancing automation (e.g., “Debug this code”) and augmentation (e.g., “Let us refine this together”) will show higher extended thinking usage.

*H4 (Socio-Technical Systems Theory)*: At the cluster level, the combination of automation (cluster_automation_index) and augmentation patterns (cluster_augmentation_index) will predict extended thinking usage (cluster_thinking_fraction).

#### 3.1.5 Hypothesis 5 (TAM X SET)

Technology adoption is shaped both by individual experience and by the practices for completing similar types of work. Consider how spreadsheets were adopted in organizations. An individual accountant might discover that Excel macros save hours of work. But whether she continues using macros depends partly on whether her finance team adopts them as standard practice. If colleagues start sharing macro-enabled templates and expecting automated reports, her individual discovery becomes common procedure, and that collective norm reinforces her own continued use.

H5 examines whether the task-level association between collaborative augmentation (augmentation_index) and usage intensity (task_conversation_share) operates partly through cluster-level extended thinking (cluster_thinking_fraction), a behavioral indicator of how deliberative reasoning is taken up across related tasks. Together, the TAM and SET lenses point to two influences on how intensively AI is used on a task: the task’s own interaction style, and the prevailing way AI is used across related tasks, measured here by cluster-level extended thinking. Because the data are cross-sectional, this cross-level pathway is treated as an expected association rather than a demonstrated feedback loop.

*H5 (TAM × SET)*: The relationship between task-level augmentation (augmentation_index) and task usage intensity (task_conversation_share) will be mediated by cluster-level extended thinking (cluster_thinking_fraction).

#### 3.1.6 Hypothesis 6 (SET X STS)

SET and STS theories suggest that for wide adoption of AI for a type of work, two conditions must be met: users must trust the AI enough to collaborate with it, and the AI must be effectively integrated into how work gets done. Collaborative interaction (iteration, learning, validation) should more strongly predict user adoption for work where extended thinking usage is also prevalent. This study predicts that work that needs both high collaborative interaction and high extended thinking usage will show the most widespread adoption. The combination signals that AI has been successfully integrated as a trusted thinking partner.

*H6 (SET × STS)*: At the cluster level, collaborative interaction patterns (cluster_augmentation_index) and extended thinking usage (cluster_thinking_fraction) will interact to predict broader user adoption (cluster_user_share), such that the relationship between collaborative interaction and user adoption will be stronger when extended thinking usage is high.

## 4 Procedure

### 4.1 Data sources

The Anthropic Economic Index (Handa et al., 2025; Anthropic, 2025b; Tamkin et al., 2024) dataset enables analysis of work activities at the task level and at the cluster level. This structure enables testing of hypotheses about individual task usage (H1, H2) and broader patterns of collaborative work (H3, H4, H6).

### 4.2 Analytical approach

The study tested six hypotheses using regression analysis at three levels: task level (H1, H2), cluster level (H3, H4, H6), and cross-level mediation (H5). Task-level analyses examine the intensity of AI usage for individual work activities, while cluster-level analyses examine how groups of related tasks show collaborative patterns. The cross-level analysis tests whether patterns at the task level influence outcomes at the cluster level.

#### 4.2.1 Sample sizes

- Task-level analysis: 3,365 unique tasks with complete data on key variables; one task lacked data on interaction variables and was excluded listwise from regression models resulting in *n* = 3,364
- Cluster-level analysis: Full sample of 593 clusters; analytical samples ranged from 488 to 568 clusters depending on hypothesis and variables required

The reduction from millions of conversations to thousands of tasks occurs because Anthropic aggregated individual conversations to the task level before public release. The further reduction from 3,365 tasks to 593 clusters reflects Anthropic’s semantic similarity algorithm, which assigned only 346 of the 3,365 tasks (10.3%) to cluster groupings. Within the 593 clusters, analytical sample sizes varied by hypothesis (H3: 508 clusters, H4: 488 clusters, H6: 508–568 clusters) due to different patterns of missing data on the specific variables each test required. All cluster-level analyses required complete data on extended thinking mode, a key measure of cognitive partnership.

#### 4.2.2 Dependent variables

The study measured AI usage at two levels.

Task-level usage intensity was measured by task_conversation_share, which represents the proportion of all AI conversations in the dataset that were mapped to each specific task. For example, if “Debug Python code” accounts for 2.5% of all conversations in the dataset, its task_conversation_share is 0.025. This variable ranges from 0.0015 to 6.65, with a mean of 0.0297 and standard deviation of 0.171. The distribution is heavily right-skewed, meaning most tasks show minimal AI usage while a small number of tasks account for disproportionate shares of activity. In fact, 8 tasks (0.2% of all tasks) show values exceeding 1.0, indicating they each account for more than the average share of AI usage. Because interaction patterns are observable only for tasks where AI is already in use, this measure captures usage intensity among existing uses rather than the initial decision to adopt AI, which cannot be modeled with these data.

Cluster-level usage was measured using two related indicators. The variable cluster_user_share represents the proportion of users in the dataset who engaged with tasks in that cluster. For instance, if 15% of all users had at least one conversation about software debugging tasks, that cluster’s user share is 0.15. The variable cluster_conversation_share represents the proportion of total conversations mapped to that cluster. These two measures are highly correlated (*r* = 0.995) and conceptually represent user reach (how many people use this type of work) versus usage intensity (how much total activity this type of work generates).

#### 4.2.3 Independent variables

Following Handa et al. (2025), the study used Clio’s classification of five mutually exclusive interaction patterns. Each conversation receives a single classification based on the dominant interaction type:

- *Directive*: The user delegates a task to AI with minimal back-and-forth input. Example: “Write a marketing email about our new product.”
- *Feedback loop*: The user corrects AI errors through repeated debugging cycles. Example: “Fix the syntax error in line 12… now I’m getting a different error… try adding a semicolon.”
- *Task iteration*: The user and AI collaboratively refine outputs through dialogue. Example: “Make the tone more formal… good, now add specific examples… perfect, but can you shorten it?”
- *Learning*: The user seeks understanding rather than task completion. Example: “Explain how gradient descent works” or “Why does this code approach work better than alternatives?”
- *Validation*: The user requests verification of AI outputs or their own work. Example: “Is this Python code secure?” or “Can you check if my logic is sound?”

These five patterns partition into two broader categories. Automation-oriented behaviors (directive and feedback loop) accounted for 43% of conversations. Augmentation-oriented behaviors (task iteration, learning, and validation) accounted for 57% of conversations.

At the task level, the study calculated the proportion of conversations for each task that exhibited each pattern. For example, if a task had 100 conversations and 30 involved task iteration, the task_iteration variable for that task equals 0.30. All interaction variables are proportions ranging from 0 to 1.

#### 4.2.4 Extended thinking mode

Claude 3.7 Sonnet introduced an optional “extended thinking” feature where users can request deeper deliberative reasoning before the model responds. When activated, the model generates explicit reasoning traces before producing its final answer. This feature was used in approximately 5% of conversations overall, though usage varied substantially by task type.

The study measured extended thinking at both levels. At the task level, thinking_fraction represents the proportion of conversations for that task where users activated extended thinking mode. At the cluster level, cluster_thinking_fraction represents the proportion of all conversations within that cluster that used extended thinking.

Extended thinking data were sparse at the task level, with 88% of tasks showing no recorded usage (mean = 0.058, SD = 0.035 for tasks with non-missing data). This likely occurred because extended thinking was a relatively new feature and many tasks had too few conversations to reliably estimate usage rates. By aggregating to the cluster level, where each cluster combines data from multiple related tasks, more stable estimates were achieved (mean = 0.050, SD = 0.026, with only 4.2% missing data).

Hypotheses about extended thinking (H3, H4, H6) were tested at the task cluster level. The aggregation of tasks followed this procedure: If a cluster contains three tasks with 100 conversations each, and extended thinking was used in 5 conversations for task A, 8 for task B, and 7 for task C, the cluster_thinking_fraction would be (5 + 8 + 7)/(100 + 100 + 100) = 0.067 or 6.7%.

#### 4.2.5 Composite indices

This study constructed three composite indices to capture theoretically meaningful patterns:

Automation index = directive + feedback_loop. This measures the extent to which a task or cluster involves delegating work to AI or debugging AI outputs, representing AI as a tool that executes tasks.

Augmentation index = task_iteration + learning + validation. This measures the extent to which a task or cluster involves collaborative refinement, knowledge-seeking, and verification, representing AI as a cognitive partner.

Risk management index = feedback_loop + validation. This measures behaviors that indicate users are actively managing potential errors or risks during AI use. Following PMT, tasks requiring more risk management behaviors are predicted to show lower usage intensity, as users may perceive these tasks as having higher vulnerability to AI errors or requiring more protective effort.

Each index is a simple sum of proportions. For example, if a task has directive = 0.20, feedback_loop = 0.15, task_iteration = 0.30, learning = 0.25, and validation = 0.10, then:

- Automation index = 0.20 + 0.15 = 0.35
- Augmentation index = 0.30 + 0.25 + 0.10 = 0.65
- Risk management index = 0.15 + 0.10 = 0.25

These indices were calculated at both the task level (using task-level proportions) and cluster level (using cluster-level proportions).

#### 4.2.6 Sample and measure characteristics

Missing data were handled differently at each level. At the task level, tasks with no observed AI usage were coded as zero for both usage and interaction variables. This preserves the meaningful distinction between tasks that are never performed with AI versus tasks performed with AI at low rates.

Sample sizes for cluster-level hypotheses ranged from 488 to 508 clusters depending on which variables each hypothesis required. Because testing SET and STS hypotheses requires reliable measurement of cognitive partnership, H3 used 508 clusters (85.7% of total), H4 used 488 clusters (82.3% of total), and H6 used between 508 and 568 clusters depending on the specific model tested. These variations reflect different patterns of missing data across variables rather than exclusion criteria. All cluster-level analyses used samples with complete data on extended thinking, a sample of 508 clusters, ensuring reliable measurement of cognitive partnership.

The final samples have these characteristics:

Task level (*N* = 3,365; models *n* = 3,364):

- task_conversation_share: mean = 0.0297, SD = 0.171, range = 0.0015 to 6.65
- Automation index: mean = 0.198, SD = 0.212
- Augmentation index: mean = 0.348, SD = 0.291
- Risk management index: mean = 0.0267, SD = 0.0739

Cluster level (*N* = 593 total clusters):

- cluster_user_share: mean = 0.162, range = 0.085 to 0.248
- cluster_conversation_share: mean = 0.159, range = 0.084 to 0.252
- Cluster thinking fraction: mean = 0.050, range = 0.006 to 0.161
- Cluster automation index: mean = 0.435, SD = 0.144
- Cluster augmentation index: mean = 0.563, SD = 0.140

#### 4.2.7 Hypothesis-to-variable mapping

The following table summarizes which variables were used to test each hypothesis:

Analytical methods varied by hypothesis. Task-level usage hypotheses (H1, H2) used multiple complementary approaches: ordinary least squares regression for baseline estimates, fractional logit models for probability of usage, and Gamma and Poisson pseudo-maximum-likelihood (PPML) models with log links. Cluster-level hypotheses (H3, H4, H6) used ordinary least squares regression. Cross-level mediation analysis (H5) used a four-step approach (Baron and Kenny, 1986), with the significance of the indirect effect assessed using bias-corrected bootstrapped confidence intervals (5,000 resamples). Robust standard errors were employed throughout to account for heteroscedasticity in the highly skewed usage measures. A battery of robustness analyses, reported in full in the Supplementary material (Robustness Checks), contains details (Table 1).

**Table 1:** Variables used to test each hypothesis.

Hypothesis	Theory	Level	Dependent variable	Independent variables
H1	TAM	Task	task_conversation_share	task_iteration, learning, augmentation_index
H2	PMT	Task	task_conversation_share	risk_management_index
H3	SET	Cluster	cluster_thinking_fraction	cluster_augmentation_index
H4	STS	Cluster	cluster_thinking_fraction	cluster_automation_index, cluster_augmentation_index
H5	TAM × SET	Cross-level	task_conversation_share	augmentation_index → cluster_thinking_fraction (mediation)
H6	SET × STS	Cluster	cluster_user_share	cluster_augmentation_index, cluster_thinking_fraction, and their interaction (moderation)

Each independent variable is a behavioral indicator of an actualized affordance (Strong et al., 2014), not a measure of the perceptual construct named by the corresponding theoretical lens; the constructs themselves (e.g., perceived usefulness, perceived risk, felt trust) are unmeasured.

## 5 Results

Table 2 presents the correlation matrix for interaction patterns and usage intensity. Feedback Loop showed the strongest correlation with usage intensity (*r* = 0.302, *p* < 0.001), explaining 9.1% of variance in task-level AI usage. The negative correlation between Learning and Task Iteration (*r* = −0.187, *p* < 0.001) suggests these patterns reflect different use contexts: educational exploration versus collaborative task completion.

**Table 2:** Pearson correlation matrix for interaction patterns and AI usage intensity.

Variable	1	2	3	4	5	6
1. Directive	1.000					
2. Feedback loop	0.051**	1.000				
3. Task iteration	0.473***	0.041*	1.000			
4. Learning	−0.123***	0.135***	−0.187***	1.000		
5. Validation	0.105***	0.124***	0.053**	0.028	1.000	
6. Task conversation share	0.086***	0.302***	0.078***	0.044*	0.106***	1.000

*N* = 3,365 tasks (pairwise *n* = 3,364). ***p < 0.001, **p < 0.01, *p < 0.05. Task conversation share represents AI usage intensity (percentage of conversations associated with each task).

The dominance of Feedback Loop in predicting usage intensity (*r* = 0.302) provides insight into the protective engagement mechanism. Tasks characterized by iterative error-correction show substantially higher AI usage than those involving pure delegation (*r* = 0.086) or collaborative refinement (*r* = 0.078). This suggests that risk-aware behaviors, particularly responding to environmental feedback, enable rather than suppress usage by allowing users to maintain control while building competence through repeated interaction cycles.

### 5.1 H1: technology acceptance model—task-level AI usage intensity

H1 hypothesized that tasks characterized by higher levels of iterative refinement (task_iteration) and learning-oriented interaction (learning) will show higher AI usage intensity (task_conversation_share).

To ensure robust inference across the full distribution of task_conversation_share (skewness = 23.34, with 8 tasks exceeding 1.0), this study employed three complementary approaches: an ordinary least squares (OLS) regression on the individual interaction predictors (Table 3), an OLS regression on the composite augmentation index (Table 4), and a log-linear OLS regression that models the logarithm of usage intensity to accommodate the skew (Table 5). All three produced consistent results.

Task-level OLS regression shows that both iterative refinement (*β* = 0.079, *p* < 0.001) and learning-oriented interaction (*β* = 0.040, *p* < 0.001) are positively associated with AI usage intensity (Table 3). The model explained 1.0% of variance in usage intensity, indicating that collaborative interaction is one of many factors influencing AI use patterns.

**Table 3:** Task-level AI usage intensity: individual predictors (OLS).

Variable	Coefficient	SE	*t*	*p*
Constant	0.0099	0.002	4.31	<0.001
Task iteration	0.0794	0.010	8.26	<0.001
Learning	0.0401	0.005	8.13	<0.001

Dependent variable is task_conversation_share. *F*(2, 3,361) = 103.93, *p* < 0.001; *R*^2^ = 0.010.

To assess the combined effect of interaction patterns, an augmentation index combining task_iteration, learning, and validation behaviors was constructed. The composite augmentation index (task_iteration + learning + validation) showed a positive association with task_conversation_share (*β* = 0.060, SE = 0.004, *p* < 0.001, *R*^2^ = 0.011, Table 4), consistent with the individual-predictor results.

**Table 4:** Task-level AI usage intensity: augmentation index (OLS).

Variable	Coefficient	SE	*t*	*p*
Constant	0.0085	0.002	3.89	<0.001
Augmentation index	0.0596	0.004	14.85	<0.001

Dependent variable is task_conversation_share. *F*(1, 3,362) = 220.51, *p* < 0.001; *R*^2^ = 0.011.

**Figure 1:**
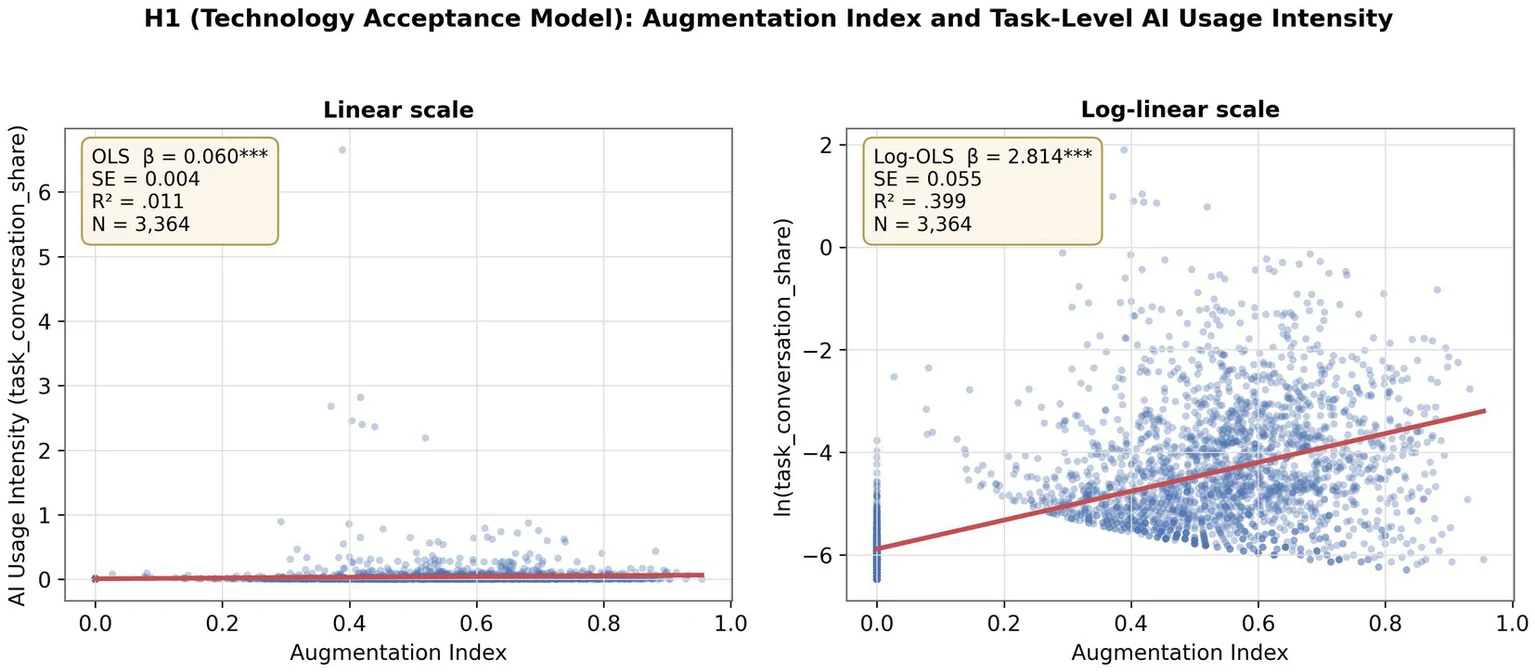
Task-level association between the augmentation index and AI usage intensity (task_conversation_share), shown on a linear scale (left) and a log-linear scale (right). Each point is a task (*N* = 3,364); red lines are OLS fits. Augmentation is positively associated with usage intensity, and the relationship is approximately exponential (log-linear *β* = 2.814, R^²^ = 0.399).

However, the substantial positive skew in task_conversation_share (skewness = 23.34, range: 0.0015–6.65), suggested a potential nonlinear relationship. To address this, a log transformation to the dependent variable (task_conversation_share) was performed and a Log-linear OLS regression showed a stronger association between the augmentation index and usage intensity (*β* = 2.814, SE = 0.055, *p* < 0.001, *R*^2^ = 0.399, Table 5). This suggests an exponential relationship: each one-unit increase in the augmentation index predicts a 16.7-fold increase in task conversation share (exp(2.814) = 16.7). Because a one-unit change spans nearly the full observed range of the augmentation index, the relationship is more meaningfully expressed per standard deviation: a one-standard-deviation increase in augmentation predicts approximately 2.3 times higher usage share (exp(2.814 × 0.291) = 2.27). The substantial improvement in model fit (*R*^2^ = 0.399) suggests that augmentation behaviors exhibit an exponential rather than linear relationship with AI usage intensity, supporting H1 (Figure 1).

**Table 5:** Log-linear OLS regression results.

Variable	Coefficient	SE	*t*	*p*
Intercept	−5.885	0.015	−389.53	<0.001
Augmentation index	2.814	0.055	50.73	<0.001

DV = log(task_conversation_share). *N* = 3,365 (models *n* = 3,364). *R*^2^ = 0.399, Adj. *R*^2^ = 0.399, *F*(1, 3,362) = 2573.56, *p* < 0.001.

### 5.2 H2: protection motivation theory—task-level AI usage intensity

Contrary to Protection Motivation Theory predictions, risk-management behaviors showed positive associations with usage intensity (H2a: *β* = 0.665, SE = 0.163, *p* < 0.001, *R*^2^ = 0.085, Table 6), indicating that tasks involving frequent error correction and output verification exhibited substantially higher rather than lower AI usage.

**Table 6:** H2a risk management index linear regression.

Variable	Coefficient	SE	*t*	*p*
Intercept	0.0115	0.002	5.46	<0.001
Risk management index	0.6649	0.163	4.09	<0.001

DV = task_conversation_share (pct). *N* = 3,365 (models *n* = 3,364). *R*^2^ = 0.085, Adj. *R*^2^ = 0.085, *F*(1, 3,362) = 16.68, *p* < 0.001.

**Figure 2:**
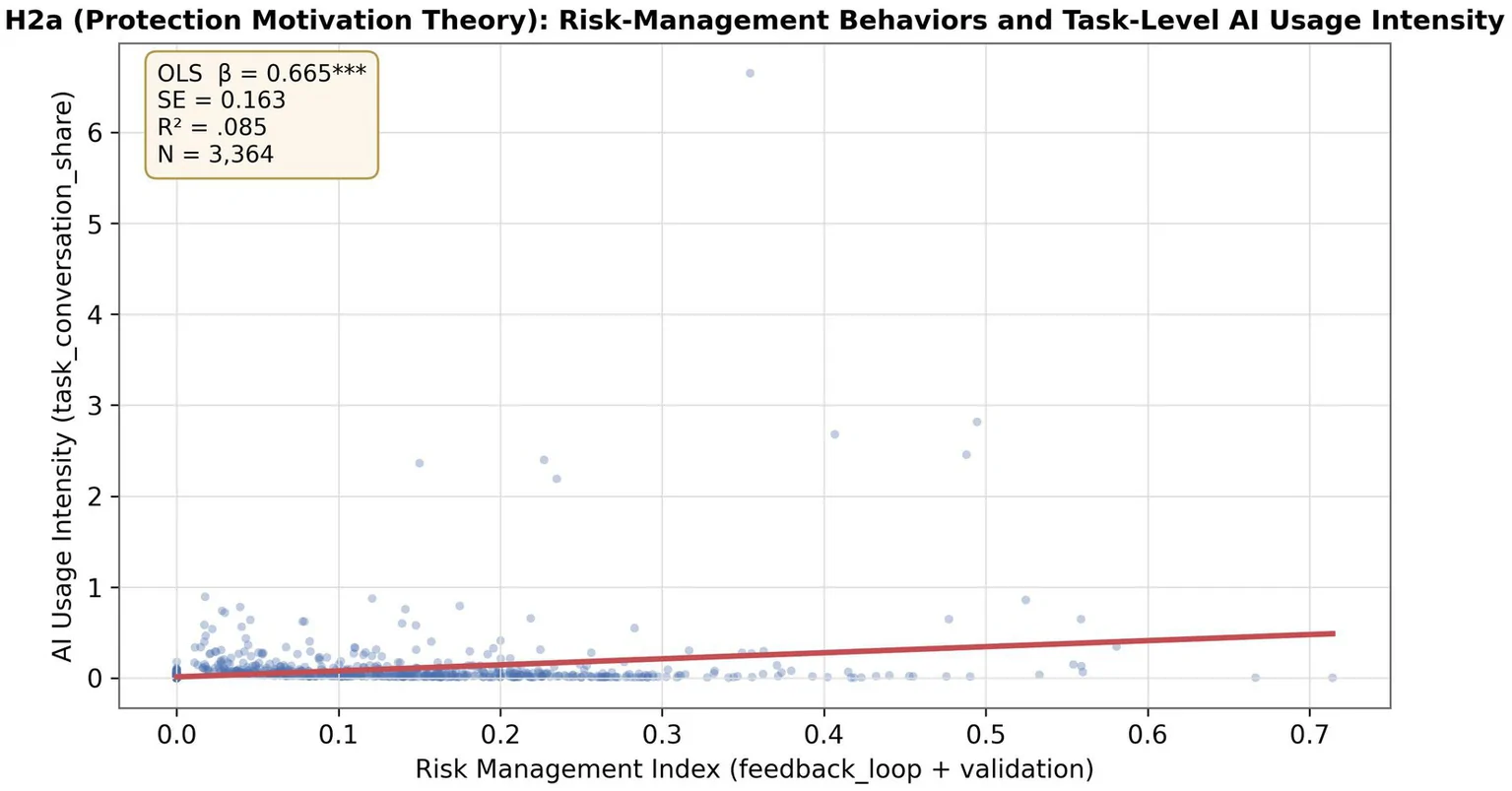
Task-level association between the risk-management index (feedback_loop + validation) and AI usage intensity (task_conversation_share). Each point is a task (*N* = 3,364); the red line is the OLS fit (*β* = 0.665, *SE* = 0.163, *R^²^* = 0.085). Contrary to PMT, risk-management behaviors are positively associated with usage intensity.

To examine whether risk-management behaviors shape engagement style beyond usage intensity, this study tested their association with automation delegation (directive) and collaborative augmentation (task_iteration). Contrary to traditional PMT predictions, risk-aware behaviors positively predicted both delegation (H2b: *β* = 0.263, SE = 0.041, *p* < 0.001) and collaboration (H2c: *β* = 0.155, SE = 0.033, *p* < 0.001), though effect sizes were small (Table 7). This suggests risk-aware monitoring enables differentiated chatbot engagement instead of dissuading chatbot use (Figure 2).

**Table 7:** H2 risk management models.

Model	Dependent variable	Coefficient	SE	*t*	*p*
H2a	Task conversation share	0.6649	0.163	4.09	<0.001
H2b	Directive	0.2630	0.041	6.39	<0.001
H2c	Task iteration	0.1553	0.033	4.65	<0.001

*N* = 3,365 tasks (models *n* = 3,364). Independent variable = risk_management_index (feedback_loop + validation). All models estimated using OLS regression.

### 5.3 H3: social exchange theory

H3 examines the SET lens using clusters of tasks to see if higher levels of collaborative augmentation (cluster_augmentation_index) will predict greater use of extended thinking mode (cluster_thinking_fraction).

**Figure 3:**
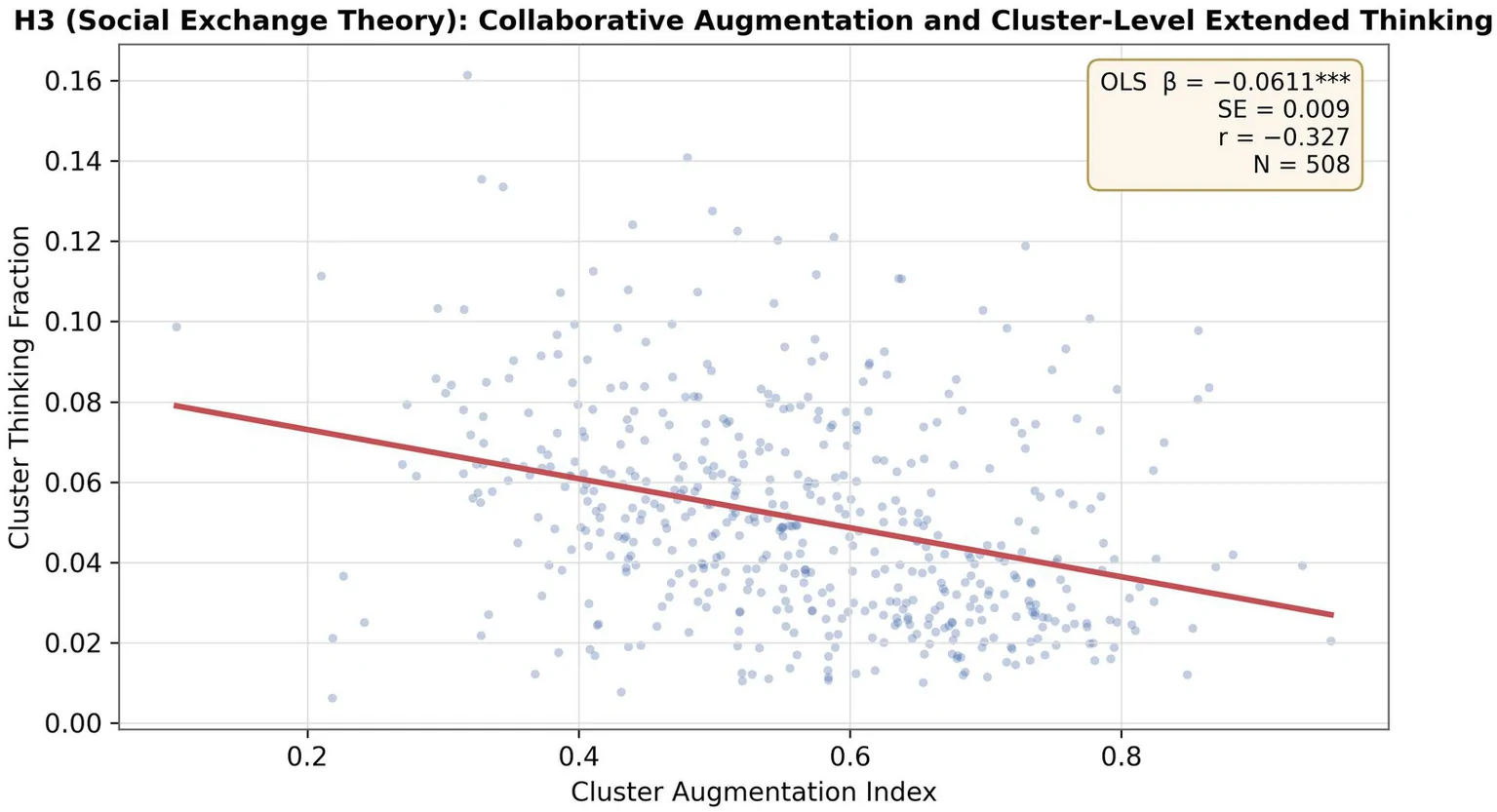
Cluster-level association between the collaborative augmentation index and extended-thinking usage (cluster_thinking_fraction). Each point is a work cluster (*n* = 508); the red line is the OLS fit (*β* = −0.0611, *SE* = 0.009, *r* = −0.327). Collaborative augmentation is negatively associated with extended-thinking usage.

Contrary to theoretical predictions, the regression analysis revealed a statistically significant negative relationship between collaborative augmentation and extended thinking usage (Table 8). As collaborative augmentation increased, extended thinking usage decreased. The model explained moderate variance in extended thinking usage (*R*^2^ = 0.107, Adjusted *R*^2^ = 0.105, *F*(1, 506) = 49.07, *p* < 0.001). This negative relationship remained robust when controlling for user breadth (*β* = −0.062, *p* < 0.001), activity volume (*β* = −0.062, *p* < 0.001), and when excluding high-influence observations (*β* = −0.076, *p* < 0.001). The estimate was also unchanged under standard errors clustered by task (*p* < 0.001). To address the possibility that extended thinking usage reflects task complexity or domain composition rather than interaction style, two additional sensitivity models were estimated with task-clustered standard errors. Controlling for technical domain, the negative augmentation-thinking association attenuated by roughly one-third but remained significant (*β* = −0.039, *p* < 0.001); technical clusters used extended thinking substantially more (*β* = 0.020, *p* < 0.001). Controlling instead for feedback-loop intensity, the association attenuated to non-significance (*β* = −0.003, *p* = 0.773). The latter adjustment should be interpreted cautiously: cluster interaction proportions sum to approximately 1.0, so the augmentation and feedback-loop measures are related (*r* = −0.62). The technical-domain model therefore provides the cleaner test of the complexity alternative, and there the negative association persists. The interpretive implications of these sensitivity analyses are taken up in the Discussion (Figure 3).

**Table 8:** H3 cluster thinking fraction linear regression.

Variable	Coefficient	SE	*t*	*p*
Intercept	0.0853	0.0052	16.55	<0.001
Cluster augmentation index	−0.0611	0.0087	−7.00	<0.001

DV = cluster thinking fraction. *n* = 508. *R*^2^ = 0.107, Adj. *R*^2^ = 0.105, *F*(1, 506) = 49.07, *p* < 0.001.

### 5.4 H4: socio-technical systems

H4 examines the STS lens at the cluster level stating that the combination of automation (cluster_automation_index) and augmentation patterns (cluster_augmentation_index) will predict extended thinking usage (cluster_thinking_fraction).

Multiple OLS regression analysis at the cluster level (*n* = 488) did not support this prediction (Table 9). When both indices were entered simultaneously, automation showed no significant relationship with extended thinking (*β* = 0.002, SE = 0.050, *p* = 0.974), while augmentation was also not significantly associated with extended thinking under heteroscedasticity-robust standard errors (*β* = −0.058, SE = 0.050, *p* = 0.244; *p* = 0.051 with task-clustered standard errors). The full model explained 10.2% of variance in extended thinking usage. At the cluster level, socio-technical alignment between automation and augmentation did not predict extended thinking usage as hypothesized.

**Table 9:** H4 multiple OLS regression analysis at the cluster level.

Variable	Coefficient	SE	*t*	*p*
Intercept	0.0836	0.048	1.73	0.083
Cluster automation index	0.0016	0.050	0.03	0.974
Cluster augmentation index	−0.0579	0.050	−1.16	0.244

DV = cluster thinking fraction. *n* = 488. *R*^2^ = 0.102, Adj. *R*^2^ = 0.098, *F*(2, 485) = 21.64, *p* < 0.001 (heteroscedasticity-robust HC3 standard errors).

### 5.5 H5: technology acceptance model X social exchange theory

H5 examined if the relationship between task-level augmentation (augmentation_index) and task usage intensity (task_conversation_share) is mediated by cluster-level extended thinking (cluster_thinking_fraction).

The mediation was estimated across the 568 task-cluster observations (328 unique tasks) for which cluster-level extended-thinking data were available. Each task is represented once for every cluster to which it belongs, so tasks spanning multiple clusters, which tend to be high-usage, carry greater weight. A statistically significant partial mediation emerged, although the mediating path ran opposite to the direction H5 predicted. The total effect of collaborative augmentation on usage intensity was negative (path c: *β* = −1.008, SE = 0.223, *z* = −4.53, *p* < 0.001, *R*^2^ = 0.031). This negative sign reflects the cluster-grain reversal reported for the correlations (cluster-level *r* = −0.119) rather than the positive task-level association estimated on the full sample (H1; *β* = 0.060, *r* = 0.103), consistent with the Simpson’s Paradox–like aggregation effect noted there.

Turning to the mediator, the path from augmentation to cluster-level extended thinking was also significant but negative (path a: *β* = −0.017, SE = 0.006, *z* = −2.70, *p* = 0.007, *R*^2^ = 0.014). Tasks exhibiting greater augmentation were embedded in clusters with lower average extended-thinking usage, contrary to the predicted direction. Because extended thinking was a newly launched feature during the observation window, this pattern indicates that intensive collaborative interaction concentrated in clusters where the feature was not yet broadly engaged.

**Figure 4:**
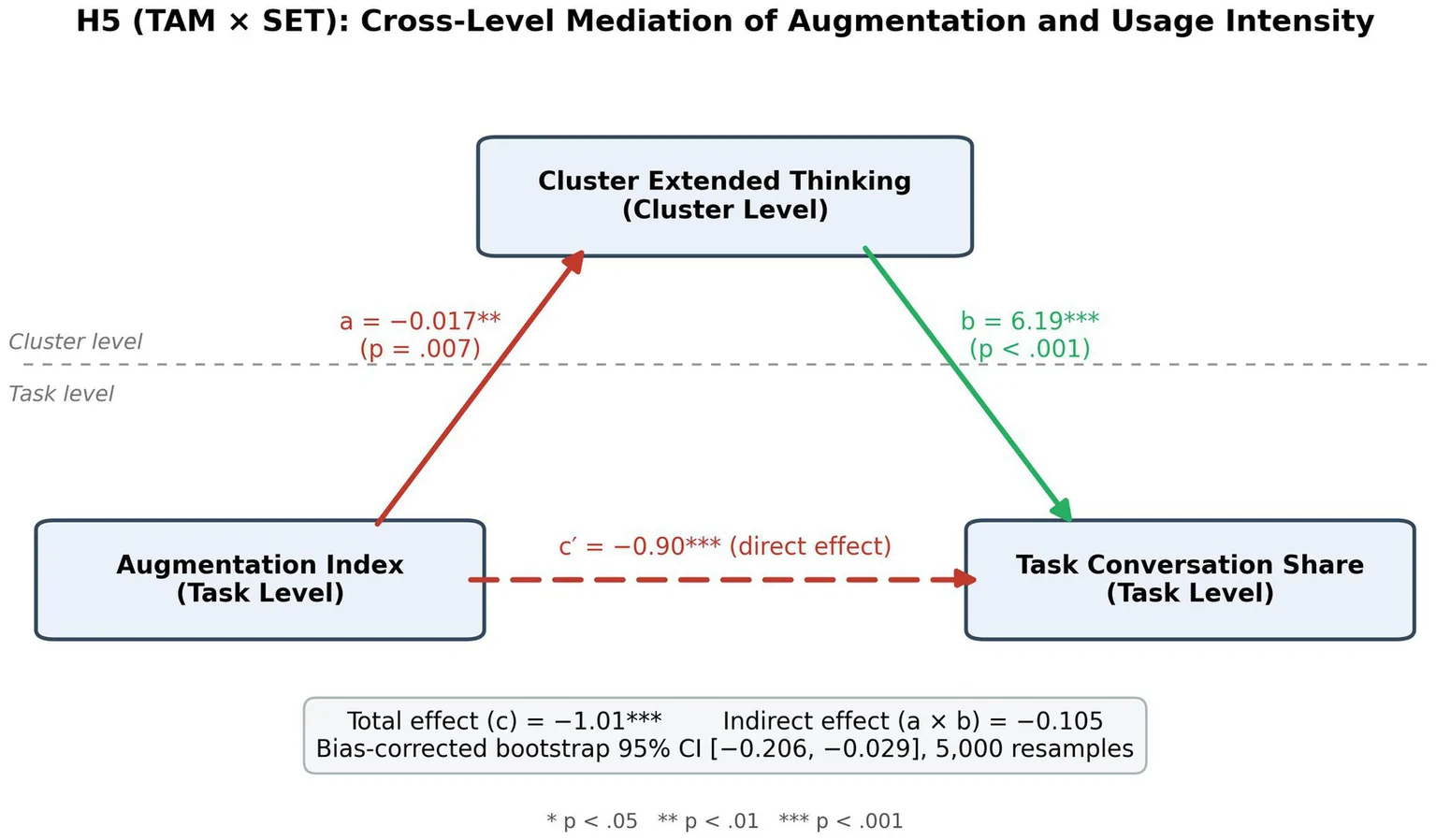
Cross-level mediation (H5) of the augmentation index and task usage intensity through cluster-level extended thinking. Path a (augmentation → cluster extended thinking) = −0.017 (*p* = 0.007); path b (cluster extended thinking → usage intensity) = 6.19 (p < 0.001); direct effect c’ = −0.90 (*p* < 0.001); total effect c = −1.01 (*p* < 0.001); indirect effect a×b = −0.105, bias-corrected bootstrap 95% CI [−0.206, −0.029], 5,000 resamples.

With both predictors entered, cluster-level extended thinking was a strong positive predictor of usage intensity (path b: *β* = 6.194, SE = 1.432, *z* = 4.33, *p* < 0.001), and the augmentation coefficient was reduced in absolute magnitude (path *c*′: *β* = −0.903, SE = 0.211, *z* = −4.28, *p* < 0.001), with improved model fit (*R*^2^ = 0.054). The indirect effect of augmentation on usage intensity through cluster-level extended thinking was −0.105, accounting for 10.4% of the total effect (−1.008 = −0.903 + (−0.105)). Bias-corrected bootstrap 95% confidence intervals excluded zero under both standard resampling [−0.206, −0.029] and task-clustered resampling [−0.262, −0.008] (5,000 resamples), confirming a reliable indirect path. This is complementary partial mediation: the path through extended thinking runs in the same direction as the direct effect and accounts for roughly a tenth of the total (Figure 4).

Together, these results support partial cross-level mediation, with the two cross-level paths running in opposite directions: augmentation is associated with less cluster-level extended thinking, while cluster-level extended thinking strongly predicts greater usage intensity. Taken together, these paths suggest that augmentation and extended thinking reflect different stages or modes of human–AI collaboration rather than redundant mechanisms. The Discussion elaborates on this interpretation.

### 5.6 H6: social exchange theory X socio-technical systems

Hypothesis 6 examined, at the cluster level, if collaborative interaction (cluster_augmentation_index) and extended thinking usage (cluster_thinking_fraction) will interact to predict broader user adoption (cluster_user_share), such that the relationship between collaborative interaction and user adoption will be stronger when extended thinking usage is high.

Aligned with Social Exchange Theory and Socio-Technical Systems Theory, work clusters combining high collaborative interaction with high extended thinking usage would demonstrate the most widespread user adoption, signaling mature socio-technical integration.

Analysis was conducted using 508 unique work clusters with complete data on all variables. User adoption share ranged from 8.5 to 24.8% (*M* = 0.162, *SD* = 0.034), representing the proportion of users engaging with AI for each type of work. Cluster augmentation, representing the combined proportion of task iteration, learning, and validation interactions aggregated to the cluster level, ranged from 10.4 to 95.5% (*M* = 0.563, *SD* = 0.140). Extended thinking fraction ranged from 0.6 to 16.1% (*M* = 0.051, *SD* = 0.026). Table 10 presents the descriptive statistics.

**Table 10:** Descriptive statistics for H6 variables (*n* = 508 clusters).

Variable	*M*	*SD*	Min	Max	Skew	Kurt
User adoption share (cluster_user_share)	0.162	0.034	0.085	0.248	0.06	−0.37
Cluster augmentation (cluster_augmentation_index)	0.563	0.140	0.104	0.955	−0.05	−0.36
Extended thinking (cluster_thinking_fraction)	0.051	0.026	0.006	0.161	0.80	0.61

Table 11 reports correlations between interaction components and AI usage across the task and cluster levels. This comparison showed several relationships that were positive at the task level reversed direction at the cluster level. Notably, the learning component showed a significant positive correlation with task conversation share at the task level (*r* = 0.044, *p* = 0.011, *N* = 3,364) but a significant negative correlation with user adoption share at the cluster level (*r* = −0.139, *p* < 0.001, *N* = 588). A Fisher’s z test confirmed that this cross-level difference was statistically significant (*z* = 4.09, *p* < 0.001).

**Table 11:** Cross-level comparison of interaction pattern correlations with AI usage.

Component	Task-level *r*	Cluster-level *r*	Direction
Directive	0.086***	0.075	Consistent
Feedback loop	0.302***	0.003	Consistent
Task iteration	0.078***	0.103*	Consistent
Learning	0.044*	−0.139***	Reversed
Validation	0.106***	−0.016	Reversed
Augmentation index	0.103***	−0.119**	Reversed

Task-level: *N* = 3,365 tasks (pairwise *n* = 3,364), DV = task_conversation_share. Cluster-level: *n* varies by component (520–593), DV = cluster_user_share. Direction indicates whether the sign of the correlation reversed across levels. *p < 0.05, **p < 0.01, ***p < 0.001.

Similarly, the composite augmentation index showed a positive correlation with usage intensity at the task level (*r* = 0.103, *p* < 0.001) but a negative correlation at the cluster level (*r* = −0.119, *p* = 0.006). This cross-level reversal is consistent with a Simpson’s Paradox–like aggregation effect, in which relationships observed at the task level reverse when examined at the system level due to heterogeneity in how interaction patterns scale across work domains. Implications of this are addressed in the Discussion.

Table 12 presents the cluster-level correlation matrix for H6 variables. Contrary to theoretical predictions, cluster augmentation was significantly negatively correlated with user adoption (*r* = −0.119, *p* = 0.006), suggesting that work clusters characterized by more collaborative interaction patterns exhibited lower rates of user adoption. Extended thinking showed essentially no relationship with adoption (*r* = −0.019, *p* = 0.649). Additionally, augmentation and extended thinking were significantly negatively correlated (*r* = −0.327, *p* < 0.001), contradicting the expectation that collaborative work would naturally incorporate sophisticated cognitive features.

**Table 12:** Cluster-level correlation matrix (*n* = 508 clusters).

Variable	1	2	3
1. User adoption share (cluster_user_share)	—		
2. Cluster augmentation (cluster_augmentation_index)	−0.119**	—	
3. Extended thinking (cluster_thinking_fraction)	−0.019	−0.327***	—

*n* = 508 clusters ***p < 0.001, **p < 0.01, *p < 0.05.

A hierarchical regression tested H6 with mean-centered predictors and heteroscedasticity-consistent (HC3) standard errors (VIF = 1.12 for both predictors). Table 13 presents the results across four models. In Model 1, cluster augmentation alone significantly predicted user adoption, but in the negative direction (*B* = −0.027, *p* = 0.012, *R*^2^ = 0.013). Extended thinking alone (Model 2) did not significantly predict adoption (*B* = −0.005, *p* = 0.933, *R*^2^ < 0.001).

**Table 13:** Hierarchical regression results predicting user adoption.

Predictor	Model 1	Model 2	Model 3	Model 4
Intercept	0.177***	0.162***	0.182***	0.162***
Augmentation	−0.027*	—	−0.031**	−0.031*
Extended thinking	—	−0.005	−0.060	−0.065
Aug × Thinking	—	—	—	−0.273
*R* ^2^	0.013	0.000	0.014	0.015
Adjusted *R*^2^	0.011	−0.002	0.011	0.010
*F*	6.30*	0.01	3.49*	2.45

*n* = 508 clusters. *F*(3,504) = 2.45, *p* = 0.063. Entries are unstandardized coefficients; significance based on heteroscedasticity-consistent (HC3) standard errors. Predictors mean-centered for the interaction. *p < 0.05, **p < 0.01, ***p < 0.001.

When both predictors were entered simultaneously (Model 3), only augmentation remained significant (*B* = −0.031, *p* = 0.008; *β* = −0.127), while extended thinking remained non-significant (*B* = −0.060, *p* = 0.386; *β* = −0.046). Adding extended thinking did not improve model fit (Δ*R*^2^ = 0.002).

Testing the interaction term in Model 4, the augmentation × extended thinking interaction was not significant (*B* = −0.273, *p* = 0.570), and adding the interaction did not improve model fit (Δ*R*^2^ = 0.001). The full model explained only 1.54% of the variance in user adoption (*R*^2^ = 0.015, *F*(3, 504) = 2.45, *p* = 0.063).

Hypothesis 6 was not supported as predicted. The positive main effects of both collaborative augmentation and extended thinking on system-level adoption were not observed; instead, augmentation showed a significant negative relationship with adoption. The hypothesized interaction, where high collaboration combined with high extended thinking would amplify adoption, was not significant.

The cross-level reversal shown in Table 11 suggests that the positive relationship between augmentation and usage intensity observed at the task level (*r* = 0.103) does not aggregate to the cluster level (*r* = −0.119). This suggests that while individual tasks with more collaborative interactions may attract more AI usage, work *clusters* dominated by collaborative patterns show lower overall user adoption. The negative association was significant but small: a one-standard-deviation increase in cluster augmentation corresponded to a 2.4% relative decrease in user adoption (Table 13, Model 1).

## 6 Discussion

This study analyzed millions of human–AI conversations from the Anthropic Economic Index to examine how chatbot interactions with Claude AI are associated usage are associated with usage across occupational tasks. As lenses that generate expectation about post-adoptive technology use, four frameworks (TAM, PMT, SET, and STS) examined usage intensity of AI as a cognitive partner. The analysis explored collaborative augmentation, risk-management behaviors, extended thinking, and their roles across job tasks and clusters of job activities. The results are mixed. Some expectations hold, others do not, and the cross-level consistency, from tasks to clusters, expected under the socio-technical lens is contradicted.

### 6.1 Hypothesis 1

Hypothesis 1 was supported: among tasks where AI is already used, those involving iterative refinement and knowledge-seeking interactions showed higher usage intensity. Treated as a lens on this post-adoptive behavior, TAM’s expectation is borne out with learning-oriented exchange and iterative control as the behavioral counterparts of its drivers.

These findings extend prior descriptive work (Handa et al., 2025; Anthropic, 2025b) by showing that augmentation is positively associated with how intensively AI is used on a task. Among tasks where AI is already used, higher levels of collaborative interaction are associated with disproportionately greater usage: a one-standard-deviation increase in collaborative interaction corresponds to 2.3 times the usage intensity (Table 5). Collaborative interaction appears to function as a post-adoption amplifier, deepening sustained use.

Through the TAM lens, the pattern is consistent with AI being used more where it proves useful for understanding complex work and easy to steer through back-and-forth interaction, with iteration offering control and learning-oriented exchange signaling value. Because the data record behavior rather than perceptions, these remain post-adoptive interpretations that tasks used as a back-and-forth thinking partner carry more usage than one-shot query tasks.

An important limitation is that this analysis includes only tasks with observed AI usage, since collaborative interaction patterns are only measurable for tasks already adopted. The current study characterizes the relationship between collaborative interaction and usage intensity, rather than modeling the initial adoption decision itself. Future research combining behavioral data with task characteristics such as complexity and skill requirements could more directly examine what drives initial adoption.

A practical implication for leaders: AI use concentrates in work where the system is used as a thinking partner. Tasks that involve iterative refinement, clarification, and learning-oriented interaction show consistently higher levels of AI use. In other words, people use AI most readily when it helps them think through problems, not just do work for them. Because the relationship is multiplicative, the same shift toward collaborative interaction yields a larger absolute gain in tasks where usage is already high than in tasks where it is low. For organizations, this suggests that encouraging iterative, learning-oriented interaction may deepen usage more effectively than simply widening access to AI tools.

### 6.2 Hypothesis 2

The findings for Hypothesis 2 challenge a common PMT and managerial assumption, that concern about risk slows AI use. The results point the other way. Tasks where users engage in frequent error-checking (feedback loops) and output verification (validation) showed higher usage intensity. This contradicts the prediction that risk-aware behaviors would suppress usage and is consistent with risk-management behaviors functioning as protective engagement (Fu et al., 2025).

This extends PMT beyond binary adoption decisions. With ongoing AI use, risk perception shapes how much users engage, supporting flexibility in applying AI to tasks. The same users who monitor AI closely are more likely to delegate routine tasks when appropriate and collaborate more on complex or ambiguous work. Here risk awareness expands users’ options, developing a practical understanding of where AI performs well and where it needs oversight. It suggests that users who validate and iterate through feedback are actively building competence and calibrating appropriate trust.

Risk management is associated more with how intensively AI is used than with which interaction pattern dominates. The link to usage intensity is considerably stronger than the links to delegation or iterative collaboration, which are positive but small. The value of risk-aware behavior lies in supporting how intensively AI is used rather than in steering the choice between delegating and collaborating.

This protective-engagement interpretation should be held alongside a simpler alternative. Feedback loops and validation tend to concentrate in complex, error-prone tasks, and such tasks also attract heavier use, so part of the positive association may reflect task complexity rather than risk management enabling engagement. Future research may evaluate if risk-management behavior still predicts usage intensity once complexity is accounted for.

For organizations, these findings suggest that encouraging verification and iterative feedback may support rather than hinder AI use. Training that teaches users to check AI outputs and give corrective feedback may help them calibrate trust and sustain productive use across diverse tasks.

### 6.3 Hypothesis 3

The disconfirmation of Hypothesis 3 reveals an unexpected pattern in human–AI collaboration with chatbots. Task clusters characterized by higher levels of collaborative augmentation exhibited lower rates of extended thinking usage. Several interpretations of these findings are possible. First, that collaborative interaction and extended autonomous reasoning are not complementary expressions of trust, but through which users structure effective partnerships with AI systems. Second, that extended thinking concentrates in technically complex, debugging-intensive work, so the negative association may partly reflect what tasks demand rather than how users choose to express trust.

Rather than indicating a failure of SET, the observed negative relationship between collaborative augmentation and extended thinking usage suggests that trust in AI systems may have different interaction pathways:

- Delegation pathway: Human steps back, lets AI think autonomously (extended thinking)
- Collaboration pathway: Human stays actively engaged, guides iteratively (augmentation)

In the delegation pathway, users express trust by allowing the AI to operate autonomously on complex tasks. Requests for extended thinking appear most frequently on these tasks, where users delegate responsibility for reasoning depth and accept longer response times in exchange for more comprehensive analysis. Activating extended thinking where a task exceeds what a simpler exchange can handle and a longer, more autonomous pass is worth the wait. Here, extended thinking would function as a behavioral marker of reliance on the AI’s reasoning, though the current data cannot confirm the underlying trust.

In the collaboration pathway, users stay engaged in shaping outputs through iterative dialogue, distributing the cognitive effort across the human and the AI rather than concentrating it in the system. Extended thinking becomes less necessary here, because the reasoning is already being guided step by step.

These pathways may represent different expressions of trust. Users who develop collaboration trust find iterative dialogue productive enough that extended autonomous reasoning would slow rather than help the partnership. Users who develop delegation trust accept stepping back in exchange for deeper independent analysis.

This distinction helps explain why collaborative augmentation and extended thinking usage are negatively associated at the cluster level. High-augmentation contexts are not characterized by lower trust or lower sophistication; rather, they reflect a different configuration of cognitive labor. When users are deeply engaged in co-creation, autonomous extended reasoning may be perceived as disruptive to work or unnecessary given ongoing human guidance.

Extended thinking is used much more in technical domains. Controlling for technical domain cut the negative association between augmentation and extended thinking by about a third, and controlling for feedback-loop intensity removed it. Here, extended thinking serves partly as a fallback for hard problems, so its negative association with augmentation may reflect what the work demands rather than, or alongside, a trust choice. Current data cannot fully test the two pathways; both fit the observed pattern, and they may operate jointly, with task demands shaping where extended thinking helps and trust pathways shaping how users engage it. Tracking the same clusters over time, as extended thinking matures, is an opportunity for future research.

These findings challenge the assumption that adoption of advanced AI features necessarily signals more mature or sophisticated use. Collaborative augmentation, through sustained iteration, refinement, and contextual guidance, represents a highly developed form of human–AI partnership even when extended thinking is rarely used. Sophistication here is in interaction, not necessarily using the extended thinking feature. Treating feature adoption as a linear indicator of trust or acceptance risks mischaracterizing user behavior. Users may rationally avoid certain features not due to lack of trust, but because their preferred interaction renders those features irrelevant.

For researchers, the dual-pathways suggest an addition to how Social Exchange Theory applies to human–AI collaboration by showing that trust in AI can shape which process users choose, not just how much they engage. Unlike human-human collaboration, where greater trust typically accompanies delegation of harder work, human–AI collaboration allows users to express trust by selecting between fundamentally different interaction styles. Both pathways reflect partnerships of qualitatively different types.

For organizations, the findings suggest supporting multiple collaboration modes rather than promoting a single best way to use AI. Designing procedures that help users recognize, select, and transition between pathways based on task may better support long-term human–AI collaboration. Training programs that push all users toward task delegation may disadvantage those whose tasks and thinking styles work better with collaboration. Metrics focused merely on feature adoption may miss the partnerships developing through iterative dialogue.

### 6.4 Hypothesis 4

Hypothesis 4 was not supported. STS predicted that clusters exhibiting joint optimization of automation and augmentation would show higher extended thinking usage, reflecting alignment between technical capabilities and human work practices. Instead of reinforcing one another, the two operated independently: automation showed no association with extended thinking usage, and the negative association of augmentation seen under H3 was not statistically significant in the combined model.

Rather than the joint optimization STS anticipates, which assumes performance improves, the data show that when users engage in rich iterative dialogue they draw less on the AI’s autonomous reasoning. This challenges traditional applications of STS, which assume performance improves as more technical capabilities are available. In human–AI collaboration, effective alignment may not mean activating every advanced feature at once; users appear to settle into stable configurations that fit their tasks, some relying on iterative collaboration, others on autonomous reasoning when collaboration is impractical.

For leaders, this suggests a practical lesson: more features are not necessarily associated with better integration. What matters is how well users match interaction mode to task demands, not how many advanced capabilities they use at once. Mature socio-technical systems are not those that use every feature, but those that know when to use which, so AI tools that let users move between autonomous processing and collaborative dialogue may outperform those built as one-size-fits-all.

### 6.5 Hypothesis 5

Hypothesis 5 examined whether cluster-level extended thinking mediates the relationship between task-level augmentation and task usage intensity. The mediation was statistically significant but operated in an unexpected direction: augmentation was negatively associated with cluster-level extended thinking, which in turn positively predicted usage intensity. Because the indirect path through extended thinking and the direct path were both negative, the indirect effect reinforced rather than offset the direct relationship, accounting for about a tenth (10.4%) of the total effect. In contrast to H1, this model is estimated across task-cluster pairs rather than tasks alone; its negative total and direct paths reflect the Simpson’s Paradox–like reversal reported earlier.

Part of this may be a matter of timing. Extended thinking launched in February 2025 with Claude 3.7 Sonnet and was observed only days into its availability (Anthropic, 2025b). The negative association between augmentation and extended thinking may reflect early-stage use. Clusters with established collaborative augmentation were familiar workflows, while exploratory use of extended thinking concentrated in some technical domains such as software development. Augmentation-intensive clusters may represent relatively mature human–AI collaboration with little immediate need for a newly released autonomous-reasoning feature, which could explain why augmentation and extended thinking appear negatively associated at the cluster level. An opportunity for future research is to track how the relationship evolves as the feature and familiarity matures.

With the TAM and SET lenses, H5 findings suggests the two do not operate as expected: the usefulness-driven engagement TAM emphasizes feeds the autonomous extended reasoning SET would treat as trust only weakly, and in the opposite direction, so the two appear to describe largely distinct modes of post-adoptive use rather than links in one chain. Both modes still contribute to use, but through different routes: augmentation supports iterative co-creation, while extended thinking supports delegated deep reasoning, although, as with H3, task complexity may partly determine where extended thinking concentrates. For practitioners, effective AI use is less about choosing the right interaction mode and more about matching modes to tasks and stages of maturity, treating collaboration and delegation as complementary tools.

### 6.6 Hypothesis 6

Hypothesis 6 asked whether collaborative augmentation and extended thinking would combine to predict broader adoption. However, no interaction was found, and augmentation predicted adoption negatively rather than positively. These findings suggest that neither Social Exchange Theory’s trust-building mechanisms nor Socio-Technical Systems Theory’s socio-technical alignment principles operate as predicted at the cluster level.

For leaders, this means that monitoring collaboration intensity or extended thinking usage will not necessarily show which work will see broad AI uptake. The drivers of adoption breadth appear to operate independently of the interaction patterns observable in individual conversations. Scaling AI use, then, may require more than promoting sophisticated features: widespread uptake may depend more on organizational decisions, infrastructure access, and task suitability than on how individuals interact with the tool.

### 6.7 Limitations

Because no attitudinal data were collected, independent variables are viewed as a behavioral indicator of an actualized affordance rather than as a measure of the construct that motivated it. The dependent measures speak to the intensity of post-adoptive chatbot use, clarified with each hypothesis. All measures are aggregates over tasks and clusters, so the findings describe task- and cluster-level patterns and cannot inform about individual users. The data are also cross-sectional and observational, so the associations reported here describe co-occurrence and cannot establish causal direction.

## 7 Conclusion

The findings show that effective human–AI collaboration does not follow a single path. Collaborative interaction goes with deeper AI use within tasks, while risk-aware behaviors accompany flexible, sustained engagement rather than withdrawal. Advanced features such as extended thinking play a narrower role, used mainly when simpler chat reaches its limits in specialized occupations. Not every expectation held: at the cluster level, collaboration-heavy work was associated with less extended thinking and narrower adoption, a cross-level reversal the traditional frameworks did not anticipate.

Together, these results suggest that AI use is best understood as a multi-level process shaped by interaction design, task demands, and user judgment. For researchers, they show the value of studying real interaction behavior: depth and breadth of use are governed by multiple factors. Users also appear to engage in more than one way, delegating reasoning to the AI in some work and guiding it through iterative dialogue in others, a distinction consistent with different trust pathways, though not the only explanation the data allow. Both are potential ways of working productively with AI.

For leaders, if the goal is organization-wide adoption, the implication is to support diverse ways of working with AI rather than a single prescribed approach. The quality of the human–AI working relationship, not AI capability alone, may shape whether these tools strengthen human work. Scaling use and deepening use pose distinct challenges, and successful integration may mean supporting both.

## Data Availability

The original contributions presented in the study are included in the article/Supplementary material, further inquiries can be directed to the corresponding author.
