# Correction: The collaboration code: how humans and AI work together across millions of conversations on job tasks

**DOI:** 10.3389/frai.2026.1961460

**Published:** 2026-09-02

**Authors:** Frontiers Production Office

**Affiliations:** Frontiers Media SA, Lausanne, Switzerland

**Keywords:** augmentation and automation, generative AI adoption, human–AI collaboration, protection motivation theory, social exchange theory, socio-technical systems, technology acceptance model, post-adoptive behavior

The Figures 1, 4 were in the wrong order in the PDF/XML version of this paper. Figure 4 caption and corresponding image should be changed to Figure 1 and Figure 1 caption and corresponding image should be changed to Figure 4, as shown below. The order has now been corrected.

**Figure 1:**
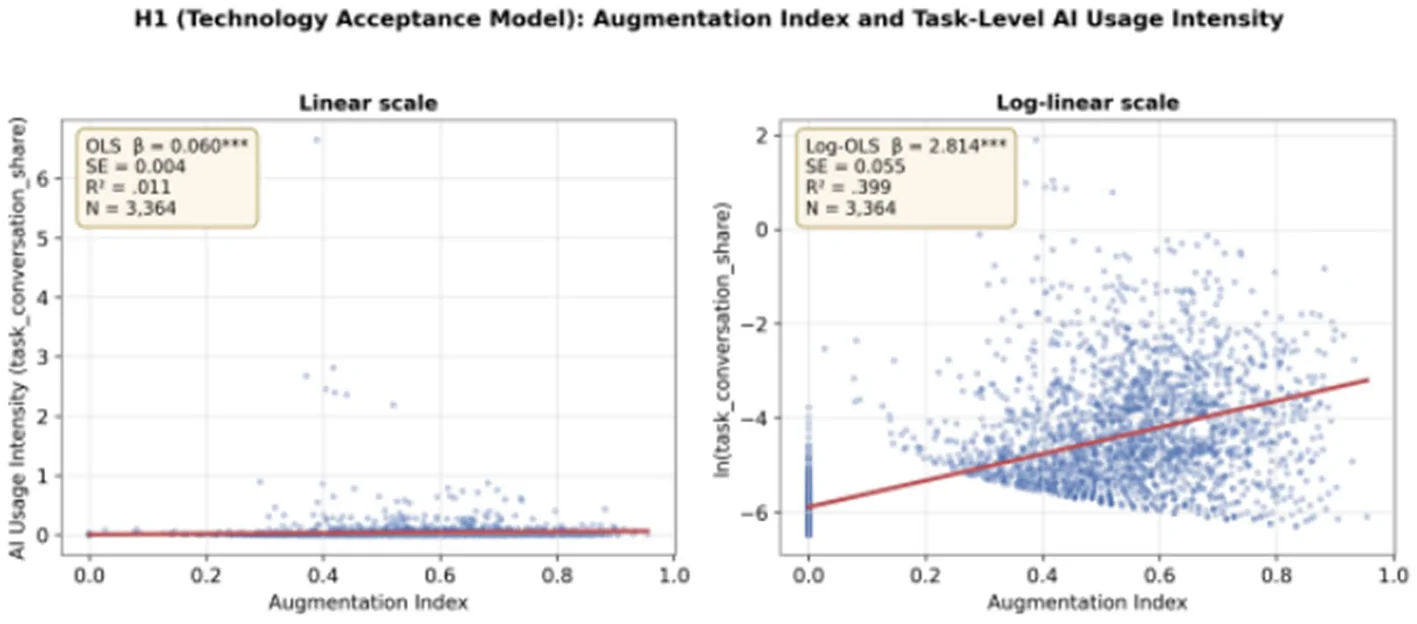
Cross-level mediation (H5) of the augmentation index and task usage intensity through cluster-level extended thinking. Path a (augmentation → cluster extended thinking) = −0.017 (*p* = 0.007); path b (cluster extended thinking → usage intensity) = 6.19 (*p* < 0.001); direct effect *c’* = −0.90 (*p* < 0.001); total effect c = −1.01 (*p* < 0.001); indirect effect *a* × *b* = −0.105, bias-corrected bootstrap 95% CI [−0.206, −0.029], 5,000 resamples.

**Figure 4:**
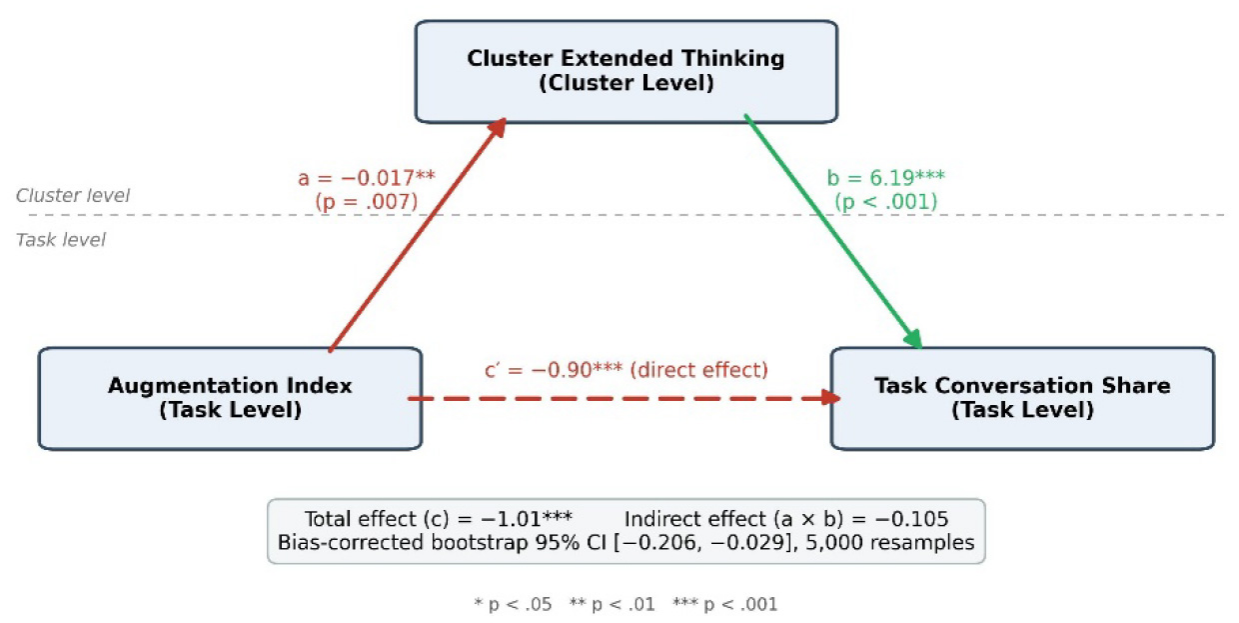
Task-level association between the augmentation index and AI usage intensity (task_conversation_share), shown on a linear scale (left) and a log-linear scale (right). Each point is a task (N = 3,364); red lines are OLS fits. Augmentation is positively associated with usage intensity, and the relationship is approximately exponential (log-linear β = 2.814, *R*^2^ = 0.399).

The original version of this article has been updated.

## Generative AI statement

Any alternative text (alt text) provided alongside figures in this article has been generated by Frontiers with the support of artificial intelligence and reasonable efforts have been made to ensure accuracy, including review by the authors wherever possible. If you identify any issues, please contact us.

