# Supplementary material for: The collaboration code: how humans and AI work together across millions of conversations on job tasks
Source: Front Artif Intell. 2026 Jul 14;9:1801235. doi: 10.3389/frai.2026.1801235 (PMC13408262; doi:10.3389/frai.2026.1801235)
Supplement: Supplementary file 1 [file Supplementary_file_1.docx]

# Data Sources and Dataset Construction

This study analyzed publicly available data from the Anthropic Economic Index (Anthropic, 2025b), hosted on HuggingFace (<https://huggingface.co/datasets/Anthropic/EconomicIndex>). The source data derive from approximately 1 million anonymized conversations between Claude.ai users (Free and Pro tiers) and Claude 3.7 Sonnet, collected between December 2024 and March 2025. Conversations were classified using Clio, a privacy-preserving methodology that maps natural language exchanges to standardized occupational tasks (Tamkin et al., 2024). In validation tests, Clio achieved 90.7% agreement with expert human raters on a sample of 150 conversations (Handa et al., 2025). Each conversation was assigned to one of approximately 19,500 tasks from the U.S. Department of Labor's O*NET occupational taxonomy (National Center for O*NET Development, 2025) and classified into one of five mutually exclusive interaction patterns: directive (complete task delegation), feedback loop (iteration driven by system errors or environment feedback), task iteration (collaborative human-AI refinement), learning (knowledge acquisition and explanation-seeking), and validation (verification of human-completed work). Following Handa et al. (2025), the first two patterns characterize automation-oriented use (43% of conversations), while the latter three characterize augmentation-oriented use (57% of conversations). In addition, the March 2025 release introduced measurements of extended thinking mode, a Claude 3.7 Sonnet feature that enables deeper cognitive processing when explicitly activated by the user.

The March 2025 release also included a bottom-up taxonomy of semantic usage clusters derived from unsupervised learning on conversation embeddings. Unlike the O*NET mapping, which imposes a top-down occupational structure, these clusters represent emergent patterns of actual use—naturally occurring categories of tasks that users bring to the system. Clusters are organized hierarchically across three levels of granularity: 593 primary clusters (e.g., "Solve geometry and trigonometry problems"), 143 intermediate clusters (e.g., "Solve advanced mathematical problems with detailed explanations"), and 30 broad clusters (e.g., "Solve scientific and mathematical problems with explanations"). Each cluster includes aggregated interaction ratios, extended thinking usage rates, and adoption metrics indicating the share of users and conversations within that cluster.

### Dataset Construction

A multi-level research dataset was constructed by integrating four source files from the March 2025 release, using the normalized task description (task_name) as the common merge key (see Table 1 for source file specifications). Three task-level files were joined to form a base of 3,365 O*NET tasks with observed Claude usage: task prevalence data (task_pct_v2.csv), interaction pattern ratios (automation_vs_augmentation_by_task.csv), and extended thinking fractions (task_thinking_fractions.csv). Extended thinking data were available for 620 of these tasks (18.4%), reflecting the proportion of O*NET tasks for which users activated this mode during the observation period.

Cluster-level data were then joined from the cluster_level_dataset directory using a left join on task_name, preserving all task-level rows. Of the 3,365 tasks, 346 appeared in Anthropic's cluster taxonomy. Because the clustering methodology permits a single O*NET task to map to multiple semantic clusters—for example, "administer, proctor, or score academic or diagnostic assessments" maps to both "Help with course enrollment, attendance, and exam administration" and "Create or implement academic assessment and scoring systems"—94 tasks were assigned to more than one cluster, yielding 593 task-cluster mappings. This expanded the dataset from 3,365 to 3,612 rows: 3,019 rows representing tasks without cluster assignments (one row per task) and 593 rows representing task-cluster combinations.

Three composite indices were computed at both the task and cluster levels: an automation index (directive + feedback loop), an augmentation index (task iteration + learning + validation), and a risk management index (feedback loop + validation). The risk management index operationalizes Protection Motivation Theory as risk-aware interaction during AI use rather than pre-adoption threat avoidance, consistent with recent reframings of PMT for generative AI contexts (Shrivastava, 2025). When any component ratio was privacy-suppressed, the corresponding composite index was treated as missing rather than zero; Anthropic’s Clio system withholds interaction ratios below approximately 0.5% to protect user privacy, and these values appear as missing in the source data. Complete variable definitions and coding specifications are provided in the data dictionary (Supplementary Material).

### Analytical Samples

The multi-level dataset structure yields distinct analytical samples for each hypothesis family. Task-level hypotheses (H1 and H2), which test Technology Acceptance Model and Protection Motivation Theory predictions about adoption and risk-aware engagement, draw on the 3,365 unique O*NET tasks; the 94 tasks assigned to multiple clusters were deduplicated to their task-level values, which are identical across cluster assignments. Because one task carried missing interaction components, the task-level regression models were estimated on 3,364 complete cases. Cluster-level hypotheses (H3, H4, and H6), which test Social Exchange Theory and Socio-Technical Systems Theory predictions about trust emergence and system-level adoption, draw on the 593 task-cluster combinations. The effective regression samples are smaller because each model drops, through listwise deletion, any row with a privacy-suppressed (missing) value in a model variable: H3 was estimated on 508 clusters, H4 on 488 (fewer because it requires both the automation and augmentation composite indices, either of which may be missing), and H6 on 508. The cross-level mediation hypothesis (H5) examines how task-level collaborative interaction relates to adoption through cluster-level extended thinking and was estimated on 568 task-cluster observations spanning 328 unique tasks. Table 2 summarizes the analytical sample, dependent variable, and independent variables for each hypothesis.

### TABLE 1

**Table 1.** Source data files from the Anthropic Economic Index March 2025 release and their contribution to the integrated research dataset.

| **Source File** | **Contents** | **Key Variables** | **Records** | **Role in Dataset** |
| --- | --- | --- | --- | --- |
| task_pct_v2.csv | Task prevalence | task_name, pct | 3,365 | Base file; adoption proxy |
| automation_vs_augmentation_by_task.csv | Interaction pattern ratios | directive, feedback_loop, task_iteration, learning, validation, filtered | 3,365 | Interaction patterns |
| task_thinking_fractions.csv | Extended thinking usage | thinking_fraction | 620 | Trust indicator (task level) |
| cluster_level_dataset/ | Semantic cluster assignments, cluster-aggregated interaction ratios, adoption metrics | cluster_name_0/1/2, cluster_thinking_fraction, percent_users, percent_records | 593 mappings (346 unique tasks) | Cluster-level variables |

Note: All files merged on task_name. Source data available under CC-BY license at <https://huggingface.co/datasets/Anthropic/EconomicIndex>.

### TABLE 2

**Table 2.** Hypothesis-to-data mapping showing analytical level, sample, and variable operationalization for each hypothesis.

| **Hypothesis** | **Theory** | **Level** | **n** | **DV** | **IV(s)** |
| --- | --- | --- | --- | --- | --- |
| H1 | TAM | Task | 3,364 | pct | task_iteration, learning, augmentation_index |
| H2 | PMT (reframed) | Task | 3,364 | pct | risk_management_index |
| H3 | SET | Cluster | 508 | cluster_thinking_fraction | cluster_augmentation_index |
| H4 | STS | Cluster | 488 | cluster_thinking_fraction | cluster_automation_index, cluster_augmentation_index |
| H5 | TAM × SET | Cross-level | 568 | pct | augmentation_index → cluster_thinking_fraction |
| H6 | SET × STS | Cluster | 508 | percent_users | cluster_augmentation_index, cluster_thinking_fraction |

Note: TAM = Technology Acceptance Model; PMT = Protection Motivation Theory; SET = Social Exchange Theory; STS = Socio-Technical Systems Theory. DV = dependent variable; IV = independent variable. Variable pct was named task_conversation_share for this manuscript. Per-hypothesis n is the analytical sample after listwise deletion of rows with privacy-suppressed (missing) values in a model variable; the constructed cluster dataset comprises 593 task-cluster mappings. H5 spans 568 task-cluster observations across 328 unique tasks. The variable percent_users corresponds to cluster_user_share (user adoption share) in the manuscript.

### Robustness Checks

**Model specification for a skewed dependent variable.** The task-level dependent variable, task_conversation_share, is a continuous, strongly right-skewed share rather than a count, so count models such as the negative binomial are not appropriate. To confirm that the H1 result does not depend on the ordinary least squares specification, the task-level usage relationship was re-estimated under five specifications that handle skew in different ways: OLS on the raw share, log-linear OLS, a fractional logit model, a Gamma generalized linear model with a log link, and a Poisson pseudo-maximum-likelihood (PPML) model with a log link. The augmentation index is a positive and highly significant predictor under every specification (Table 3). Information criteria are not directly comparable across the raw and log scales, so the back-transformed root mean squared error is reported as a common-scale comparison; it is nearly identical across models.

### TABLE 3

Goodness-of-fit comparison for the task-level usage model (H1). Predictor = augmentation index; n = 3,364. RMSE is back-transformed to the raw-share scale for comparability.

| **Model** | **Coefficient** | **(Pseudo-) R2** | **AIC** | **BIC** | **RMSE (raw)** |
| --- | --- | --- | --- | --- | --- |
| OLS (raw share) | 0.060*** | 0.011 | -2468.2 | -2456.0 | 0.1676 |
| Log-linear OLS | 2.814*** | 0.399 | 9590.7 | 9603.0 | 0.1681 |
| Fractional logit | 2.302*** | 0.099 | 19.6 | 31.9 | 0.1679 |
| Gamma GLM (log link) | 4.396*** | 0.303 | -6694.0 | -6681.8 | 0.1710 |
| Poisson PPML (log link) | 2.301*** | 0.099 | 821.9 | 834.2 | 0.1679 |

Note. *** p < .001. Coefficients are on each model’s native scale and are not directly comparable across rows; all are positive and significant.

**Exclusion of high-influence tasks.** The original submission reported 62 tasks with a share above 1.0; that figure was an artifact of a pre-deduplication dataset and does not describe the analytic sample. In the corrected complete-case sample (n = 3,364), 7 tasks have a share above 1.0. Excluding them (n = 3,357) leaves the task-level conclusions essentially unchanged: the H1 log-linear augmentation coefficient is 2.804 (versus 2.814 on the full sample), the H1 components remain significant (task iteration 0.067, learning 0.041, both p < .001), the fractional-logit estimate is 2.822, and the H2a risk-management coefficient is 0.306 (p < .001). At the cluster level, excluding 35 high-influence observations identified by Cook’s distance (threshold 4/n) the negative association between cluster augmentation and extended-thinking usage persists and strengthens (b = -0.076, p < .001, n = 473).

**Non-independence and clustered standard errors.** Because 346 tasks are distributed across 593 cluster observations, with 94 tasks appearing in more than one cluster, the cluster-level and cross-level tests were re-estimated with standard errors clustered by task (Table 4). Significance is unchanged for every test. For the H5 cross-level mediation, the indirect effect was assessed with bias-corrected bootstrap confidence intervals under both independent and task-clustered resampling (5,000 resamples); both exclude zero (independent [-0.206, -0.029]; task-clustered [-0.262, -0.008]).

### TABLE 4

Key coefficients under heteroscedasticity-consistent (HC3) and task-clustered standard errors.

| **Test** | **Coefficient** | **b** | **p (HC3)** | **p (clustered)** |
| --- | --- | --- | --- | --- |
| H3 | cluster augmentation | -0.061 | <.001 | <.001 |
| H4 | cluster augmentation | -0.058 | .244 | .051 |
| H6 | cluster augmentation | -0.031 | .008 | .008 |
| H6 | interaction (augmentation by thinking) | -0.273 | .570 | .541 |

**Alternative explanations for extended-thinking usage (H3).** Reviewers noted that extended-thinking usage may reflect task complexity rather than a trust pathway. The H3 model was therefore re-estimated with proxies for technical content and debugging intensity. Adding a technical-domain indicator reduces the augmentation coefficient by roughly a third (from -0.061 to -0.039, p < .001), with the indicator itself strongly positive; adding feedback-loop intensity renders the augmentation coefficient non-significant (-0.003, p = .77). These results indicate that extended-thinking usage is partly a function of what the work demands, consistent with the dual task-demand and trust reading retained in the Discussion.

### Reproducibility

All source data are publicly available from the Anthropic Economic Index under a CC-BY license. Dataset construction and all analyses are fully reproducible from the replication materials accompanying this submission. The build script (01_build_dataset.py) downloads the four source files, performs the merges and deduplication described above, preserves privacy-suppressed values as missing, and computes the composite indices; the analysis script (02_analysis.py) estimates H1 through H6 with HC3 heteroscedasticity-consistent standard errors; and two further scripts provide the model-comparison and sensitivity analyses reported in the manuscript. The build script writes a verification log reporting row counts and SHA-256 checksums so that replication output can be confirmed against the values reported here. The complete repository is provided to the editor and reviewers with this submission and will be made publicly available upon acceptance.

### REFERENCES

Anthropic. (2025b). Anthropic Economic Index [Data set]. Hugging Face. <https://huggingface.co/datasets/Anthropic/EconomicIndex>

Handa, K., Carlin, B., Dragan, A., Ganguli, D., Grosse, R., Goodman, N., et al. (2025). Which economic tasks are performed with AI? Evidence from millions of Claude conversations. Anthropic Research. <https://assets.anthropic.com/m/2e23255f1e84ca97/original/Economic_Tasks_AI_Paper.pdf>

National Center for O*NET Development. (2025). O*NET OnLine. <https://www.onetonline.org/>

Shrivastava, P. (2025). Understanding acceptance and resistance toward generative AI technologies: A multi-theoretical framework integrating functional, risk, and sociolegal factors. Front. Artif. Intell. 8:1565927. doi: 10.3389/frai.2025.1565927

Tamkin, A., Askell, A., Lovitt, L., Durmus, E., Joseph, N., Kravec, S., et al. (2024). CLIO: Privacy-preserving insights into real-world AI use. arXiv preprint arXiv:2410.13265.
